# Association between fecal incontinence and cardiovascular disease in adult Americans: evidence from NHANES 2005–2010

**DOI:** 10.3389/fcvm.2024.1447913

**Published:** 2024-10-17

**Authors:** Chenkai Xu, Yongfu Song, Yuejiao Lan, Yongji Wang, Na Wang, Xiaodan Lu

**Affiliations:** ^1^ College of Integrative Chinese and Western Medicine, Changchun University of Chinese Medicine, Changchun, Jilin, China; ^2^ Precision Medicine Center, Jilin Province General Hospital, Changchun, Jilin, China; ^3^ Department of Pediatrics, The Affiliated Hospital to Changchun University of Chinese Medicine, Changchun, Jilin, China

**Keywords:** fecal incontinence, cardiovascular disease, Americans, cross-sectional study, NHANES

## Abstract

**Objectives:**

There is limited amount of research on the association between fecal incontinence (FI) and cardiovascular disease (CVD). This study aims to evaluate whether there is a relationship between FI and CVD among adults in the United States.

**Methods:**

This study employed a cross-sectional design, encompassing 11,237 adults aged 20 years and older, drawn from the National Health and Nutrition Survey conducted from 2005 to 2010. FI was defined as the involuntary monthly leakage of solid, liquid, or mucus stool. The presence of CVD was evaluated through a questionnaire. Adjusted odds ratios (OR) were computed utilizing a multivariate logistic regression model. Subgroup analyses were conducted to ascertain the stability of the results.

**Results:**

Following adjustments for population characteristics, lifestyle habits, laboratory tests, and comorbidities, a significant association was observed between FI and elevated CVD risk (OR: 1.47, 95% CI: 1.21–1.79, *P* < 0.001). Subgroup analysis uncovered a strong correlation between FI and CVD among participants aged 45–65 years (OR: 1.78, 95%CI: 1.31–2.43). In the participants to aged 66 and above, this correlation persisted (OR: 1.31, 95% CI: 1.01–1.70).

**Conclusions:**

This study reveals a significant positive correlation between FI and CVD. Middle-aged and older adults are considered high-risk population for developing CVD, thus emphasizing the importance of screening and timely intervention.

## 1 Introduction

The global prevalence of cardiovascular disease (CVD) exceeds 607.6 million cases and continues to escalate, making it a prominent contributor to approximately 19 million annual deaths worldwide. In the United States, CVD stands out as an exceptionally lethal condition, responsible for nearly one-third of all deaths (1). According to statistics from 2014 to 2015, the economic burden associated with these conditions in the United States (US) was estimated to be approximately $351.2 billion (1). Therefore, the implementation of screening and early intervention for CVD is of paramount importance. One of the crucial elements in screening and intervention involves identifying risk factors associated with CVD and ascertaining high-risk populations.

Fecal incontinence (FI) is a prevalent health condition affecting individuals across genders and age groups. Epidemiological data from American communities estimate the prevalence of FI to be approximately 8.3% among the population (2) The loss of autonomous control over bowel movements imposes a significant social and economic burden on patients, ultimately leading to a notable deterioration in their quality of life. On average, each patient bears an annual economic cost as high as $4,110 due to this condition (3). These figures underscore the significant impact of this disease on both the national healthcare system and the economy. Despite the high prevalence of FI and the serious health burden imposed by CVD, there remains a dearth of research exploring the potential relationship between these two conditions. Existing studies have primarily focused on the traditional risk factors for CVD, such as hypertension, obesity, and smoking (4), with limited attention given to gastrointestinal disorders. While existing research suggests that certain digestive system disorders, such as inflammatory bowel disease and gallstones, can increase the risk of CVD (5, 6), the association between FI and CVD remains largely unexamined, particularly in the US population. This knowledge gap emphasizes the necessity for a thorough investigation into the potential relationship between FI and CVD.

The present study made use of data collected by the National Health and Nutrition Examination Survey (NHANES) over three research cycles, spanning from 2005 to 2010. The dataset encompassed extensive information related to demographics, dietary interviews, laboratory tests, and comorbidities. The primary aim of this study was to investigate the correlation between FI in American adults and CVD. Additionally, the study sought to determine the consistency of this correlation in subgroup analyses, with the ultimate goal of pinpointing high-risk populations for CVD within the FI population.

## 2 Manuscript formatting

### 2.1 Data source

The primary objective of this research is to examine the correlation between FI and CVD. NHANES, a nationally representative survey project devised and executed by the National Center for Health Statistics (NCHS), is aimed at assessing the health and nutritional status of the non-institutionalized population in the United States in a comprehensive manner. Conducted biennially, this survey offers in-depth interviews to approximately ten thousand participants, thereby providing an extensive insight into national health and nutrition. This study strictly adhered to ethical guidelines and obtained ethical approval from the NCHS Institutional Review Board (IRB). All participants provided informed consent after fully understanding the study's purpose. Furthermore, this cross-sectional study adheres to the Strengthening the Reporting of Observational Studies in Epidemiology (STROBE) guidelines, thereby ensuring transparency and scientific rigor in the research methodology (7).

**Figure 1:**
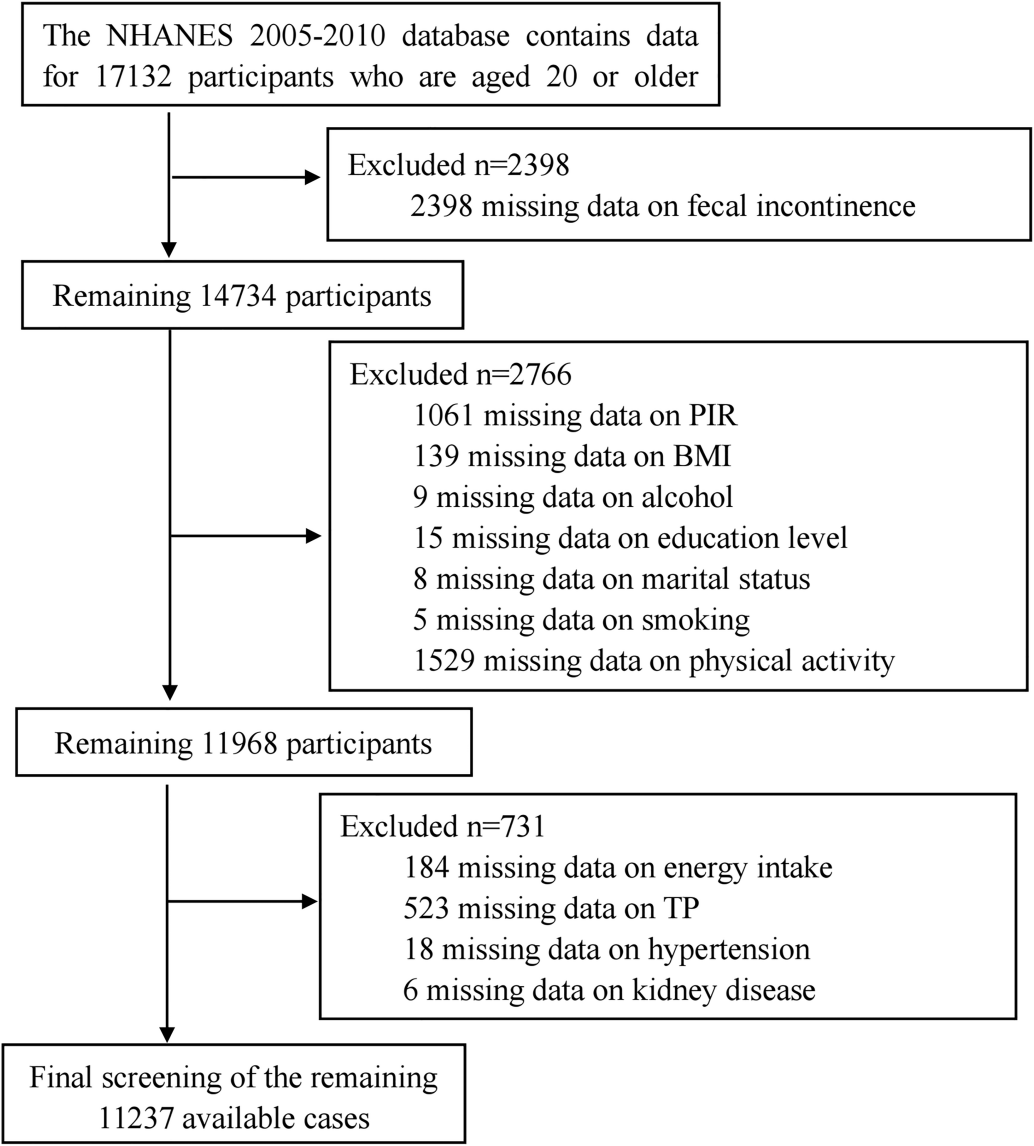
Flow diagram of the sample selection from the national health and nutrition examination survey (NHANES) 2005–2010.

The data for this study were obtained from the NHANES database, specifically from three research cycles conducted from 2005 to 2010, which included dedicated questionnaires on gut health. The study focused on adults aged 20 and above. Exclusions were made based on incomplete gut health and CVD data, as well as inadequate demographic information, dietary data, laboratory tests, and comorbidities. The exclusion process is outlined in Figure 1, resulting in a final sample size of 11237 participants.

### 2.2 Fecal incontinence assessment

The FI severity index is used in gut health surveys to assess the severity of unintentional bowel leakage, including leaks of solid stool, gas, liquid, and mucus, over the preceding month (8). This index categorizes occurrences based on their frequency: never occurred, 1–3 times per month, weekly or more frequently, twice or more weekly, daily or more frequently, and twice or more daily. FI denotes any involuntary loss of mucus, liquid, or solid stool that has occurred within the past 30 days. To facilitate analysis, we will categorize the fecal incontinence data from the NHANES database into two distinct groups: absence of occurrence (no) and any frequency of occurrence (yes) (9).

### 2.3 Cardiovascular disease assessment

We obtained data on cardiovascular disease from the medical status questionnaire within the NHANES database. The questionnaire includes the following questions: Have you ever been diagnosed by a doctor or other healthcare professional with congestive heart failure, coronary artery disease, angina, heart attack, or stroke? If any of these questions are answered affirmatively, the respondent is considered to have CVD, otherwise, they are considered not to have CVD.

### 2.4 Covariates

We extracted fundamental demographic data regarding the participants from the database, including gender (male, female), age (continuous variable), race/ethnicity (Mexican American, other Hispanic, non-Hispanic white, non-Hispanic black, other race), marital status (widowed/divorced/separated/never married, married/living with partner), education level (<high school, completed high school, >high school), body mass index (BMI) (BMI ≥ 30 kg/m^2^ for obesity, BMI ≥ 25 kg/m^2^ and BMI < 30 kg/m^2^ for overweight, BMI < 25 kg/m^2^ for normal weight), poverty income ratio (PIR) (PIR < 1 for poor, PIR ≥ 1 for not poor), and physical activity (inactive, moderate, vigorous). Smoking status is classified into three categories: never smoked, former smoker, and current smoker. Never smoked refers to individuals who have never smoked in their lifetime or have smoked fewer than 100 cigarettes. Former smokers are defined as those who have smoked more than 100 cigarettes in their lifetime but currently ceased smoking. Current smokers are individuals who have smoked more than 100 cigarettes in their lifetime and continue to smoke. Alcohol consumption was defined as drinking more than 12 alcoholic beverages per year, whereas no alcohol consumption referred to having fewer than 12 drinks per year.

We obtained pertinent dietary data, encompassing energy, fat, and protein intake, through dietary interviews conducted by NHANES. If data were available for both days of the dietary interview, we calculated the average values for those days. In the event that data for the second day were missing, we used the data from the first day as the average value. Additionally, we performed laboratory tests to gather participants’ blood biochemical indicators, specifically total protein (TP). Detailed procedures for measuring these biochemical indicators are available on the NHANES official website. The comorbidities associated with our study include diabetes, kidney disease (renal failure and kidney stones), and hypertension.

### 2.5 Statistical analyses

We conducted a descriptive analysis of the data supplied by all participants. Based on the characteristics of the data, continuous variables were analyzed using measures like the mean and standard deviation (SD), or the median and interquartile range (IQR). Categorical variables were presented as percentages (%). Categorical variables were compared using chi-square tests, while continuous variables were analyzed via *t*-tests. Logistic regression models were utilized to investigate the association between FI and CVD. No covariates were adjusted in Model I. In Model II, adjustments were made for demographic factors including gender, age, race/ethnicity, marital status, education level. Model III further incorporated adjustments for lifestyle-related covariates such as smoking status, alcohol consumption, physical activity, PIR, and BMI. Model IV includes the covariates from Model III, as well as dietary fiber intake, energy intake, fat intake, and TP. In Model V, adjustments were made for the covariates from Model IV, as well as comorbidities including hypertension, diabetes, and kidney disease. Subgroup analyses were performed to evaluate the stability of the association between FI and CVD, considering factors such as gender, age groups, BMI, smoking status, physical activity, diabetes. Statistical significance was ascertained by comparing the adjusted odds ratios (OR) with their respective 95% confidence intervals (CI). All analyses were carried out using the statistical software package R (provided by The R Foundation, accessible at http://www.R-project.org) and Free Statistics software version 1.9.2. Two-tailed tests were utilized, wherein a *P*-value less than 0.05 denoted statistically significant differences.

## 3 Results

### 3.1 Baseline characteristics

This study comprised 11,237 participants from the NHANES cycles spanning 2005–2006, 2007–2008, and 2009–2010. Among these participants, a total of 993 individuals were diagnosed with FI, yielding a prevalence rate of 8.8%. Table 1 presents a summary of the basic characteristics of the participants. FI was predominantly observed among females, older individuals, those identifying as non-Hispanic white, physically inactive individuals, well-educated and wealthy individuals, those who consume alcohol, and obese individuals, who may have a higher risk of developing FI. Among the total cohort, 1,132 participants were identified with CVD, yielding a prevalence rate of 10.1%. Compared to those without FI (9.1%), individuals with FI demonstrated a notably elevated risk of CVD (20.3%).

**Table 1:** Data characteristics of the participants.

Variables	Total *N* = 11,237	Without FI *N* = 10,244	With FI *N* = 993	*P*-value
Gender, *n* (%)				<0.001
Male	5,560 (49.5)	5,120 (50)	440 (44.3)	
Female	5,677 (50.5)	5,124 (50)	553 (55.7)	
Age, mean ± SD	48.9 ± 17.7	48.0 ± 17.7	57.8 ± 16.1	<0.001
TP, mean ± SD	7.1 ± 0.5	7.2 ± 0.5	7.1 ± 0.5	0.002
Energy intake, mean ± SD	2,069.1 ± 873.5	2,072.6 ± 877.5	2,032.5 ± 831.1	0.167
Fat intake, mean ± SD	77.2 ± 40.1	77.2 ± 40.1	78.2 ± 40.7	0.450
Protein intake, mean ± SD	80.9 ± 36.6	81.2 ± 36.6	78.3 ± 36.3	0.018
Marital status, *n* (%)				<0.001
Married/living with partner	6,962 (62.0)	6,400 (62.5)	562 (56.6)	
Widowed/divorced/separated/never married	4,275 (38.0)	3,844 (37.5)	431 (43.4)	
Race/ethnicity, *n* (%)				<0.001
Mexican American	1,921 (17.1)	1,794 (17.5)	127 (12.8)	
Other Hispanic	949 (8.4)	883 (8.6)	66 (6.6)	
Non-Hispanic White	5,831 (51.9)	5,235 (51.1)	596 (60)	
Non-Hispanic Black	2,086 18.6)	1,920 (18.7)	166 (16.7)	
Other Race	450 (4.0)	412 (4)	38 (3.8)	
PIR,n (%)				0.365
Poor	2,131 (19.0)	1,932 (18.9)	199 (20)	
Not poor	9,106 (81.0)	8,312 (81.1)	794 (80)	
BMI, *n* (%)				<0.001
Obesity	4,156 (37.0)	3,733 (36.4)	423 (42.6)	
Overweight	3,856 (34.3)	3,536 (34.5)	320 (32.2)	
Normal weight	3,225 (28.7)	2,975 (29)	250 (25.2)	
Education level, *n* (%)				<0.001
<high school	1,138 (10.1)	1,024 (10)	114 (11.5)	
Completed high school	1,727 (15.4)	1,537 (15)	190 (19.1)	
>high school	8,372 (74.5)	7,683 (75)	689 (69.4)	
Smoking, *n* (%)				<0.001
Never	5,951 (53.0)	5,511 (53.8)	440 (44.3)	
Former	2,882 (25.6)	2,550 (24.9)	332 (33.4)	
Current	2,404 (21.4)	2,183 (21.3)	221 (22.3)	
Physical activity, *n* (%)				<0.001
Inactive	5,102 (45.4)	4,607 (45)	495 (49.8)	
Moderate	3,190 (28.4)	2,885 (28.2)	305 (30.7)	
Vigorous	2,945 (26.2)	2,752 (26.9)	193 (19.4)	
Drink, *n* (%)				0.149
No	3,085 (27.5)	2,793 (27.3)	292 (29.4)	
Yes	8,152 (72.5)	7,451 (72.7)	701 (70.6)	
Diabetes, *n* (%)				<0.001
No	9,964 (88.7)	9,169 (89.5)	795 (80.1)	
Yes	1,273 (11.3)	1,075 (10.5)	198 (19.9)	
Hypertension, *n* (%)				<0.001
No	7,414 (66.0)	6,911 (67.5)	503 (50.7)	
Yes	3,823 (34.0)	3,333 (32.5)	490 (49.3)	
CVD, *n* (%)				<0.001
No	10,105 (89.9)	9,314 (90.9)	791 (79.7)	
Yes	1,132 (10.1)	930 (9.1)	202 (20.3)	
Kidney, *n* (%)				<0.001
No	10,208 (90.8)	9,379 (91.6)	829 (83.5)	
Yes	1,029 (9.2)	865 (8.4)	164 (16.5)	

PIR, poverty income ratio; FI, fecal incontinence; TP, total protein; BMI, body mass index; CVD, cardiovascular disease.

### 3.2 Relationship between FI and CVD

Table 2 presents the results of a univariate logistic regression analysis. The findings indicate that variables such as age, gender, race, marital status, physical activity, BMI, education level, alcohol consumption, smoking status, TP, energy intake, fat intake, protein intake, diabetes, kidney disease and FI are significantly correlated with CVD (all *P* < 0.05). The results of the logistic regression analyzing the relationship between FI and CVD are presented in Table 3. The model without adjustment for covariates (Model I) showed that increased risk of CVD was associated with FI (OR: 2.56, 95%CI: 2.16–3.03, *P* < 0.001). After adjusting for basic demographic information (Model II), FI remained significantly associated with CVD (OR: 1.71,95%CI:1.42–2.05, *P* < 0.001). After further adjustment for factors related to lifestyle habits (Model III), the association between FI and CVD remained stable (OR: 1.6,95%CI:1.33–1.93, *P* < 0.001). Further adjusted for biochemical indicators related to laboratory tests (Model IV), the significant association between FI and CVD remained (OR:1.64,95%CI:1.36–1.98, *P* < 0.001). Finally, after adjusting for all comorbidities (Model V), the outcome of FI with CVD remained stable (OR:1.47, 95%CI: 1.21–1.79, *P* < 0.001).

**Table 2:** Univariate regression analysis of the association between FI and suicidal ideation.

Variable	OR_95CI	*P*-value
Gender: female vs. Male Age	1.26 (1.1∼1.43) 1.07 (1.07∼1.08)	<0.001 <0.001
TP	0.81 (0.71∼0.92)	0.002
Energy intake	1 (1∼1)	<0.001
Fat intake	0.99 (0.99∼1)	<0.001
Protein intake	0.99 (0.99∼0.99)	<0.001
Race/ethnicity: ref.= Mexican American		
Other Hispanic	1.08 (0.78∼1.5)	0.639
Non-Hispanic White	2.29 (1.86∼2.82)	<0.001
Non-Hispanic Black	1.88 (1.48∼2.39)	<0.001
Other Race	1.04 (0.67∼1.61)	0.856
Marital status: Widowed/Divorced/Separated/Never married VS Married/living with partner	1.15 (1.02∼1.31)	0.027
Education level: ref. =<high school		
Completed high school	0.8 (0.64∼0.99)	0.04
>high school	0.56 (0.47∼0.67)	<0.001
BMI: ref. =Obesity		
Overweight	0.79 (0.69∼0.91)	0.001
Normal weight	0.58 (0.49∼0.68)	<0.001
Physical activity: ref.= Inactive		
Moderate	0.76 (0.66∼0.87)	<0.001
Vigorous	0.39 (0.32∼0.46)	<0.001
Smoking: ref.= Never		
Former	2.67 (2.32∼3.07)	<0.001
Current	1.4 (1.18∼1.66)	<0.001
Alcohol: Yes vs. No	0.84 (0.74∼0.97)	0.013
PIR: Not poor vs. Poor	0.89 (0.76∼1.03)	0.122
Diabetes: Yes vs. No	4.36 (3.78∼5.03)	<0.001
Hypertension:Yes vs. No	6.7 (5.83∼7.7)	<0.001
FI:Yes vs. No	2.56 (2.16∼3.03)	<0.001
Kidney:Yes vs. No	3.39 (2.9∼3.98)	<0.001

PIR, poverty income ratio; FI, fecal incontinence; TP, total protein; BMI, body mass index.

**Table 3:** Multivariate regression analysis of the association between FI and CVD.

Variables	Without FI OR (95%CI)	With FI OR (95%CI)	*P*-value
Model I^a^	1(Ref)	2.56 (2.16∼3.03)	<0.001
Model II^b^	1(Ref)	1.71 (1.42∼2.05)	<0.001
Model III^c^	1(Ref)	1.6 (1.33∼1.93)	<0.001
Model IV^d^	1(Ref)	1.64 (1.36∼1.98)	<0.001
Model V^e^	1(Ref)	1.47 (1.21∼1.79)	<0.001

^a^ Model I: no adjusted.

^b^ Model II: adjusted for age + gender + race/ethnicity + marital status + education level.

^c^ Model III: Model II + alcohol + smoking + physical activity + PIR + BMI.

^d^ Model IV: Model III + TP + fat intake + protein intake + energy intake.

^e^ Model V: Model IV + diabetes + hypertension + kidney.

**Figure 2:**
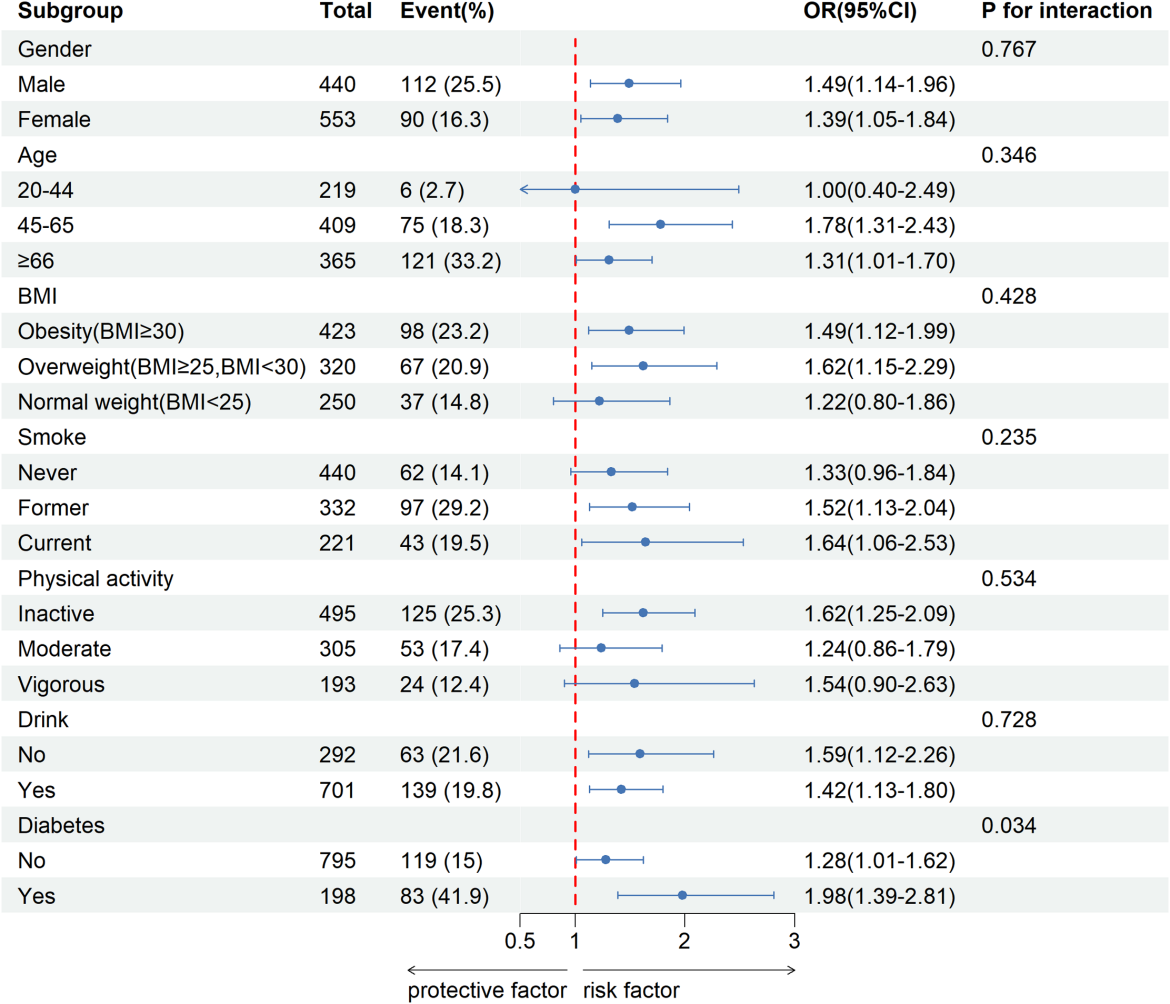
Stratified analysis of potential moderators of the relationship between fecal incontinence and cardiovascular disease.

The subgroup analysis results are presented in Figure 2. We performed subgroup analyses stratified by gender, age, BMI, smoking status, physical activity, alcohol consumption, and diabetes. The results revealed a significant interaction between diabetes and CVD (*P* < 0.05), whereas no significant interactions were observed in the other subgroups (*P* > 0.05). In the age group of 45–65, we observed a significant association between FI and CVD (OR:1.78,95%CI: 1.31–2.43). Similarly, in individuals aged 66 and older, a notable correlation was identified between FI and CVD (OR: 1.31; 95% CI: 1.01–1.70). However, no statistically significant relationship was found between FI and CVD in the younger cohort aged 20 to 44 years (OR: 1, 95% CI: 0.40–2.49).

In the sensitivity analysis, outliers in energy intake, protein intake, and fat intake were excluded, revealing a consistent association between FI and CVD (OR: 1.48, 95% CI:1.14–1.91, *P* = 0.003), as shown in Supplementary Table S1.

## 4 Discussion

This study presents several key findings: Firstly, individuals diagnosed with FI exhibit a higher propensity for developing CVD in comparison to those without FI. Secondly, irrespective of baseline characteristics and adjusted covariates, a statistically significant association persists between FI and CVD. Third, individuals aged 45 and above, especially those with FI, show a heightened susceptibility to the development of CVD. In contrast, this association is less evident in those below the age of 45.

A recent study, conducted by researchers from the United States and Japan, has revealed a significant correlation between constipation and the risk of developing CVD (10, 11). Nevertheless, the literature exploring the relationship between FI patients and CVD remains scarce, with merely a handful of studies examining this association. An observational study conducted in São Paulo has uncovered a significant correlation between elderly individuals afflicted with FI and the incidence of CVD (12). Based on a larger sample of the US population and the inclusion of numerous CVD-related covariates, our research further corroborates the robustness of the significant association between FI and CVD. Our study found that FI is still significantly associated with CVD in the elderly population. This finding is similar to the results of a study conducted in Sao Paulo.

The etiology behind the increased susceptibility to CVD linked to fecal incontinence is presumably multifaceted. Studies conducted in the United Kingdom and the Netherlands have evidenced that fecal incontinence can have substantial detrimental effects on an individual's psychological well-being, potentially inducing anxiety, shame, and depression (13, 14). Moreover, FI has the potential to cause significant physical and psychological harm. A prolonged decline in both psychological well-being and overall life quality may result in significant mental stress, subsequently leading to an elevation in blood pressure. Research has also revealed a significant correlation between fecal incontinence and hypertension (15). It is widely recognized that hypertension constitutes one of the risk factors for CVD and may underlie various observed cardiovascular conditions (16).

The gut microbiome exerts a significant influence on the pathophysiology of both FI and CVD. The microbiota-gut-brain axis, inclusive of the autonomic nervous system, facilitates bidirectional communication among the microbiota, gut, and brain (17). This intricate interaction results in the production of fecal metabolites by the gut microbiota, which subsequently modulate neurohormonal mechanisms associated with intestinal motility and sensation (18). Studies have shown that these fecal metabolites possess the capacity to augment smooth muscle contractions in the intestines and intensify neuronal activity within the enteric nervous system, ultimately manifesting as symptoms such as diarrhea, urgent defecation, and a sensation of incomplete bowel evacuation (19, 20). Furthermore, extensive research has implicated the gut microbiota in the promotion of atherosclerosis and CVD development (21–23). A mendelian randomization study has suggested that the presence of Shigella within the gut microbiome may elevate the risk of myocarditis and hypertrophic cardiomyopathy, while betaine, a gut metabolite, could heighten the risk of heart failure and myocardial infarction (24).

FI is hypothesized to play a crucial role in the pathophysiology of CVD, exerting its influence through the gut-brain-autonomic nervous system axis. Synaptic pathways within the bowel wall, responsive to sensory input, are regulated by the vagus nerve (VNS), sacral spinal nerves (S2-4), preganglionic parasympathetic fibers, and postganglionic sympathetic nerves (25). Dysfunction of the nervous system is believed to be a pivotal factor in the pathogenesis of CVD. The VNS, as the longest cranial nerve in humans, governs numerous visceral organs within the thoracic and abdominal cavities, including the heart and gastrointestinal tract (26). Compositionally, the VNS primarily consists of afferent fibers (80%) that convey sensory information from visceral organs to the brain, while the remaining efferent fibers (20%) transmit motor information to regulate adjacent organs (27, 28). The segment of the digestive tract extending from the esophagus to the splenic flexure of the colon is innervated by the parasympathetic nervous system via the vagal nerve, thereby enabling the regulation of gastrointestinal motility (29). Research has indicated that sacral nerve stimulation holds potential in improving FI and intestinal inflammation (30). The underlying mechanism is akin to cholinergic anti-inflammatory effects, facilitating the transmission of sacral nerve stimulation to colon tissue through a plausible pathway from sacral nerve input to VNS output. Sacral nerve stimulation traverses the brainstem via the spinal cord and targets organs through the VNS (31). Consequently, VNS dysfunction can significantly impact intestinal sensation and bowel function. On the other hand, the dysfunction of the nervous system is recognized as a key factor in the pathogenesis of CVD. The occurrence of FI disrupts the balance between the sympathetic and parasympathetic nervous systems, adversely affecting the heart by increasing its workload and indirectly contributing to the development of CVD (32). The VNS plays a regulatory role in myocardial cells and the conduction system of the heart, including the atria, sinoatrial node, and atrioventricular node. Additionally, postganglionic fibers from the VNS control the ventricular myocardium and conduction systems. Therefore, any impairment in vagal function could potentially impair cardiac performance (33). The interplay between the gut microbiota, fecal metabolites, and the autonomic nervous system, particularly the VNS, underscores the complex pathophysiology of FI and its potential role in the development of CVD. Further research is needed to elucidate the mechanisms and potential therapeutic interventions targeting this axis.

Our study has several limitations. Firstly, as a cross-sectional study, we cannot establish a causal relationship between FI and CVD. Secondly, limitations in the FI questionnaire data prevented the inclusion of some potential confounding variables affecting CVD. Thirdly, significant missing data on CVD in this study could potentially skew the final results.

## 5 Conclusion

This study found a significant positive correlation between FI and CVD among Americans. Our findings highlight the importance of early screening and intervention for FI patients, especially in middle-aged and older adults, in order to reduce their risk of developing CVD. Our results also indicate that FI may be an underrecognized risk factor for CVD, highlighting the need for further research to explore the underlying mechanisms linking these two conditions. Future research should give priority to prospective studies aimed at elucidating causal relationships and evaluating the efficacy of targeted interventions in reducing cardiovascular disease risk among patients with FI.

## Data Availability

Publicly available datasets were analyzed in this study. This data can be found here: https://wwwn.cdc.gov/nchs/nhanes/.
