# Supplementary material for: Association between fecal incontinence and cardiovascular disease in adult Americans: evidence from NHANES 2005–2010
Source: Front Cardiovasc Med. 2024 Oct 17;11:1447913. doi: 10.3389/fcvm.2024.1447913 (PMC11524900; doi:10.3389/fcvm.2024.1447913)
Supplement: Supplementary file 1 [file Table1.docx]

**Supplementary Table**

| **Variables** | **Without FI**  **OR (95%CI)** | **With FI**  **OR (95%CI)** | ***P*-value** |
| --- | --- | --- | --- |
| Model I^a^ | 1(Ref) | 2.74(2.196~3.43) | <0.001 |
| Model II^b^ | 1(Ref) | 1.72(135~2.21) | <0.001 |
| Model III^c^ | 1(Ref) | 1.62(1.26~2.08) | <0.001 |
| Model IV^d^ | 1(Ref) | 1.66 (1.29~2.14) | <0.001 |
| Model V^e^ | 1(Ref) | 1.48 (1.14~1.91) | 0.003 |

^a^Model I: no adjusted.

^b^Model II: adjusted for age + gender + race/ethnicity + marital status + education level + PIR + BMI.

^c^Model III: Model II **+** energy Intake + fat intake + protein intake + alcohol + smoking + physical activity.

^d^Model IV: Model III **+**TC + TG+ TP +HDL + LDL.

^c^Model V: Model IV + diabetes + hypertension + kidney.
